# Spatiotemporal expression of osteopontin in the striatum of rats subjected to the mitochondrial toxin 3-nitropropionic acid correlates with microcalcification

**DOI:** 10.1038/srep45173

**Published:** 2017-03-27

**Authors:** Tae-Ryong Riew, Hong Lim Kim, Xuyan Jin, Jeong-Heon Choi, Yoo-Jin Shin, Ji Soo Kim, Mun-Yong Lee

**Affiliations:** 1 Department of Anatomy, Catholic Neuroscience Institute, Cell Death Disease Research Center, College of Medicine, The Catholic University of Korea, Seoul, Korea; 2 Integrative Research Support Center, Laboratory of Electron Microscope, College of Medicine, The Catholic University of Korea, Seoul, Korea; 3 Gumi Electronics & Information Technology Research Institute, Gumi, Korea.

## Abstract

Our aim was to elucidate whether osteopontin (OPN) is involved in the onset of mineralisation and progression of extracellular calcification in striatal lesions due to mitochondrial toxin 3-nitropropionic acid exposure. OPN expression had two different patterns when observed using light microscopy. It was either localised to the Golgi complex in brain macrophages or had a small granular pattern scattered in the affected striatum. OPN labelling tended to increase in number and size over a 2-week period following the lesion. Ultrastructural investigations revealed that OPN is initially localised to degenerating mitochondria within distal dendrites, which were then progressively surrounded by profuse OPN on days 7–14. Electron probe microanalysis of OPN-positive and calcium-fixated neurites indicated that OPN accumulates selectively on the surfaces of degenerating calcifying dendrites, possibly via interactions between OPN and calcium. In addition, 3-dimensional reconstruction of OPN-positive neurites revealed that they are in direct contact with larger OPN-negative degenerating dendrites rather than with fragmented cell debris. Our overall results indicate that OPN expression is likely to correlate with the spatiotemporal progression of calcification in the affected striatum, and raise the possibility that OPN may play an important role in the initiation and progression of microcalcification in response to brain insults.

Osteopontin (OPN), which is an adhesive glycoprotein containing arginine-glycine-aspartate (RGD), is regarded as a multifunctional protein because it is expressed by a broad range of tissues and cells and is engaged in a variety of physiological and pathophysiological processes^1^,^2^. Accumulated evidence indicates that OPN is involved in modulating wide-ranging aspects of cellular function in the central nervous system. In particular, OPN acts as a chemoattractant that recruits microglia, macrophages, and astrocytes, thereby assisting in the formation of glial scars in response to ischaemic injury. It also has a role as a potent neuroprotectant^3^,^4^,^5^,^6^,^7^,^8^,^9^,^10^,^11^,^12^.

OPN was originally identified as a bone matrix protein^13^, and is a well-known inhibitor of bone mineralisation^14^,^15^. OPN also acts as the potent regulator of ectopic calcification and pathological mineralisation of the vasculature^16^,^17^,^18^,^19^,^20^,^21^. Recently, several lines of evidence suggest that OPN participates in the regulation of ectopic brain calcification after ischaemic insults. A previous study demonstrated that the lack of OPN induces co-occurring progressive neurodegeneration and microcalcificaton after an excitotoxic insult^22^. In addition, OPN is involved in phagocytosis of fragmented cell debris by macrophages in response to cerebral ischaemia^23^ and calcium precipitation in cell debris facilitates local binding of OPN, leading to OPN-mediated phagocytosis^24^,^25^.

It is well known that neuronal mitochondria maintain calcium ion homeostasis and their dysfunction, which may result from mitochondrial calcium overload, leads to a cascade of events that eventually end in cell death^26^,^27^,^28^,^29^. Excess mitochondrial calcium can lead to intramitochondrial calcification, which is proposed to act as calcium deposit nucleation^22^,^30^,^31^,^32^,^33^. These observations may lead us to speculate that OPN is involved in the onset of mineralisation, including in intracellular calcification, which occurs in degenerating neurons. Consistent with this hypothesis, the induction of OPN expression is associated with degenerating mitochondria in the striatal neurons of rats treated with the mycotoxin 3-nitropropionic acid (3-NP)^34^. 3-NP, which is a natural mitochondrial toxin, irreversibly inhibits the mitochondrial respiratory chain complex II, which leads to an increase in electron leakage from mitochondria and the production of reactive oxygen species^35^,^36^,^37^. Taken together, these observations indicate that the 3-NP model is a good model for better understanding the mechanisms underlying the intracellular calcification elicited by brain insults. We thus used this model to determine whether OPN is involved in the onset of intracellular calcium precipitation and subsequent extracellular calcification progression during 3-NP-induced striatal degeneration.

We examined this possibility by investigating the spatiotemporal expression profiles and subcellular localisation of OPN in the striatum of rats treated with 3-NP. In particular, we focused our attention on the relationship between OPN protein and calcium precipitation using the osmium/potassium-bichromate method to precipitate and ultrastructurally visualise endogenous calcium^38^. In addition, we analysed the shapes and spatial localisations of the OPN-labelled profiles within the lesioned striatum. We used focused ion beam milling (FIB)-scanning electron microscopy (SEM) combined with 3-dimensional (3D) reconstruction, which is a useful method for the study of brain ultrastructure and is used in correlative light and electron microscopic studies^39^,^40^,^41^.

## Results

Fifty-four animals were used in our study. We used 33 rats for immunohistochemistry (n = 6 experimental rats 1, 2, 3, 7, or 14 days after 3-NP injection, and n = 3 saline-treated control rats), 12 rats for immunoblot analysis (n = 3 controls and experimental rats 3, 7, or 14 days post-lesion), and 9 rats for immunoelectron microscopy (3, 7, or 14 days post-lesion).

### Temporal expression of osteopontin protein in 3-NP-treated rat brain

**Figure 1:**
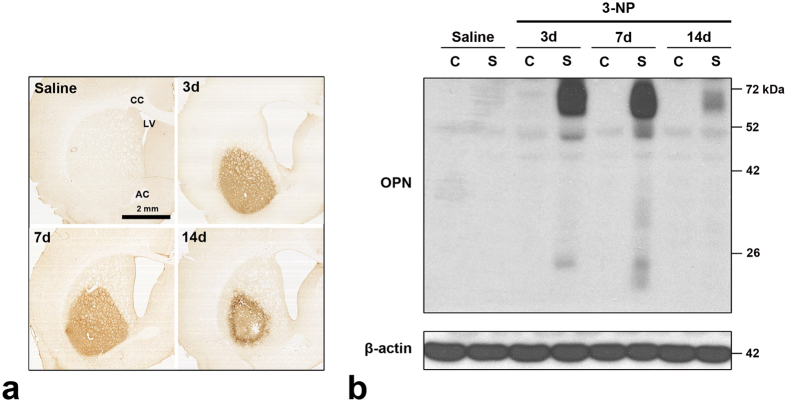
Temporal and spatial expression patterns of OPN protein in 3-NP-treated rat forebrain. (**a**) Low-magnification views of representative coronal sections from saline-treated controls and rats subjected to 3-NP treatment. No basal expression of OPN is detected in the cortex or striatum of saline-treated controls or the cortex of rats injected with 3-NP. However, there is a well-demarcated induction of OPN immunoreactivity in the lateral part of the striatum. Note that OPN expression appears to be preferentially localised to the periphery of the lesion 14 days post-injury. AC, anterior commissure; CC, corpus callosum; LV, lateral ventricle. (**b**) Immunoblotting for OPN using protein extracts from microdissected cortex (**C**) and striatum (S) of saline-treated controls and 3-NP-treated rats 3, 7, and 14 days post-lesion. OPN protein is barely detectable in the cortex and striatum of the controls and the cortex of the experimental rats, while one major band of 70 kDa and two minor bands of 50 and 25 kDa are clearly induced in striatal tissue on days 3 and 7 post-lesion. Fourteen days after 3-NP treatment, two OPN-reactive bands of 70 and 50 kDa are still observed, although they have weaker intensity. At this time, the 25 kDa band is no longer detected.

We used immunohistochemistry to study the cellular localisation and regional distribution of OPN protein in the striatum in rats treated with 3-NP. We observed no immunoreactivity specific to OPN when the primary or secondary antibody was omitted (data not shown). This confirmed the specificity of the OPN antibody. No significant immunoreactivity for OPN was detected in the cortex or striatum of saline-treated control rats or in the un-lesioned cortex of rats injected with 3-NP (Fig. 1a), as reported previously^3^,^4^,^5^,^23^,^25^. In contrast, administration of 3-NP caused a reproducible and well-demarcated induction of OPN immunoreactivity confined to the lateral part of the striatum (Fig. 1a). Fourteen days post-lesion, OPN expression appeared to be preferentially localised to the periphery of the striatal lesion in affected animals rather than in the core of the lesion. Enlargement of the lateral ventricle and shrinkage of the striatum were also observed.

The expression profile of OPN protein was further examined using western blot analysis of protein extracted from the cortex or striatum (Fig. 1b). Consistent with the immunohistochemical data, OPN protein was barely detectable in the cortex and striatum of control rats and in the cortex of rats injected with 3-NP. We clearly observed one major band at 70 kDa and two minor bands with apparent molecular masses of 50 and 25 kDa in striatal tissue from rats treated with 3-NP collected 3–7 days post-lesion. Fourteen days after 3-NP administration, we observed two bands reactive with the OPN antibody with molecular weights of 70 and 50 kDa, although they had weak intensity. We did not detect the approximately 25-kDa OPN band at this time point.

### Relationship between OPN and striatal lesions induced by 3-NP

**Figure 2:**
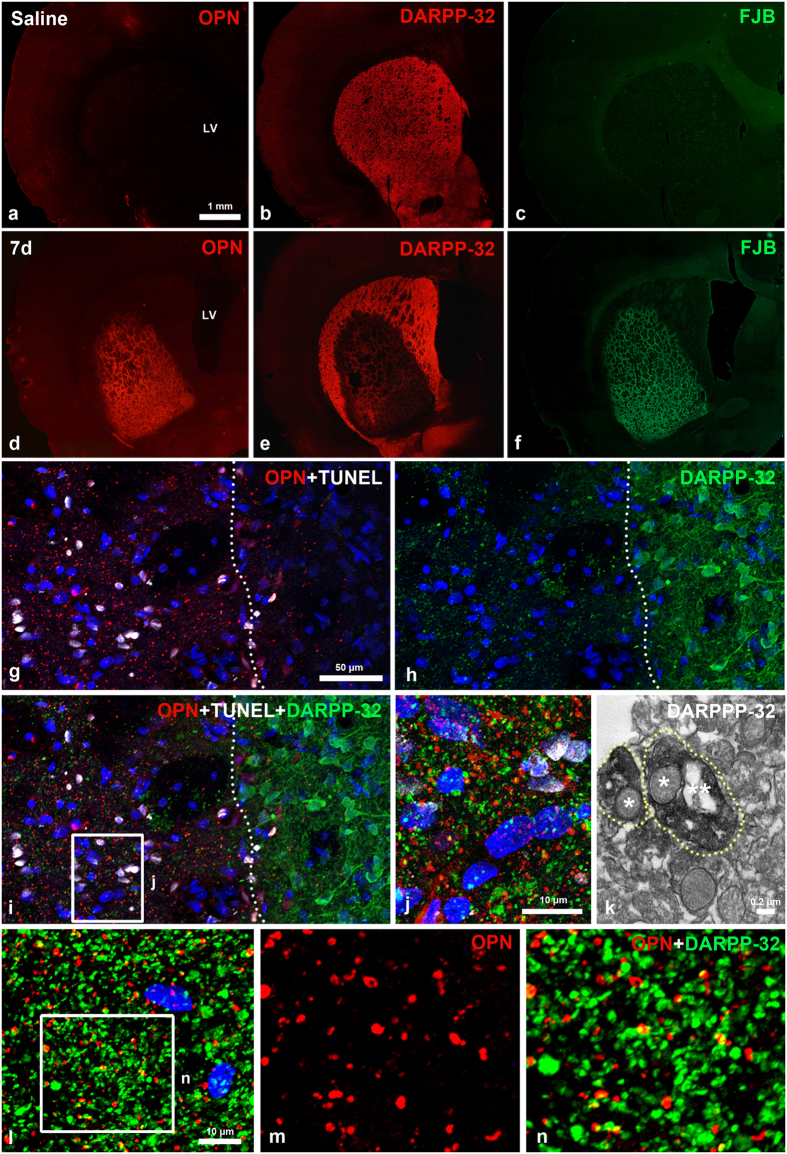
Spatial relationships between OPN and neuronal cell injury induced by 3-NP. (**a**–**f**) Serial sections of 3-NP-treated rat forebrain 7 days post-lesion are stained with OPN and dopamine- and cyclic AMP-regulated phosphoprotein with an apparent molecular weight of 32 kDa (DARPP-32; red channel), and Fluoro-Jade B (FJB; green channel). Note that a prominent OPN expression is induced within the lateral striatum, in which concomitant loss of DARPP32-positive neurons and intense FJB staining are visible in rats intoxicated with 3-NP. (**g**–**i**) Triple labelling for OPN, terminal deoxynucleotidyl transferase dUTP nick end labelling (TUNEL) and DARPP-32. OPN and TUNEL staining are restricted to the lesion core (left side of white broken line), whereas DARPP-32-positive neuronal soma with processes are evident in the perilesional area. (**j**) Higher magnification view of the lesion core (boxed area in i) showing the punctate distribution of OPN- and DARPP-32-positive profiles. (**k**) Ultrastructural features of DARPP-32-positive profiles. Dotted DARPP-32 corresponds to the immunolabelled dendrites (delimited by white dotted lines) containing the degenerating (one star) and normal-appearing (two stars) mitochondria. (**l**–**n**) Double-labelling for OPN and DARPP-32 showing that most of OPN-positive dots are co-labelled with DARPP-32, but some are devoid of DARPP32 staining, and vice versa. (m, n) Higher magnification views of the boxed area shown in i. Cell nuclei appear blue after DAPI staining.

To examine the relationship between OPN expression and neuronal cell injury induced by the 3-NP injection, we performed immunostaining on serial coronal sections from control and 3-NP–injected rats for OPN and dopamine- and cyclic AMP-regulated phosphoprotein with an apparent molecular weight of 32 kDa (DARPP-32), which is a selective neuronal marker in the striatum^42^,^43^,^44^. We also performed Fluoro-Jade B (FJB) staining, which labels degenerating neurons. No specific staining for OPN or FJB was observed in control striatum (Fig. 2a–c), where DARPP-32-positive neurons were homogeneously distributed (Fig. 2b). In rats treated with 3-NP, however, OPN expression was induced in the lateral part of the striatum (Fig. 2d), where we observed loss of DARPP32-positive neurons and intense FJB staining (Fig. 2e,f).

To determine whether the 3-NP-induced OPN protein expression was closely correlated with neuronal cell death, we performed triple-labelling for OPN, terminal deoxynucleotidyl transferase dUTP nick end labelling (TUNEL), and DARPP-32. OPN and TUNEL staining overlapped in striatal lesions, but were not detected in the perilesional area, while the opposite pattern was observed for DARPP-32 (Fig. 2g-i). Despite this complementarity, observation of the triple-stained sections at higher magnification revealed that DARPP-32 staining was visible as dots scattered throughout the lesion. This pattern was very similar to that of OPN (Fig. 2j). Subsequent immunoelectron microscopy revealed that the dot-like DARPP-32-positive pattern indeed corresponded to immunolabelled dendrites containing degenerating mitochondria (Fig. 2k). In addition, most of OPN-positive dot-like staining was co-labelled with DARPP-32, although the OPN staining corresponded to only a small fraction of the DARPP-32-labelled dots (Fig. 2l–n).

### Quantitative time-dependent analysis of OPN-positive staining in the striatal lesion

**Figure 3:**
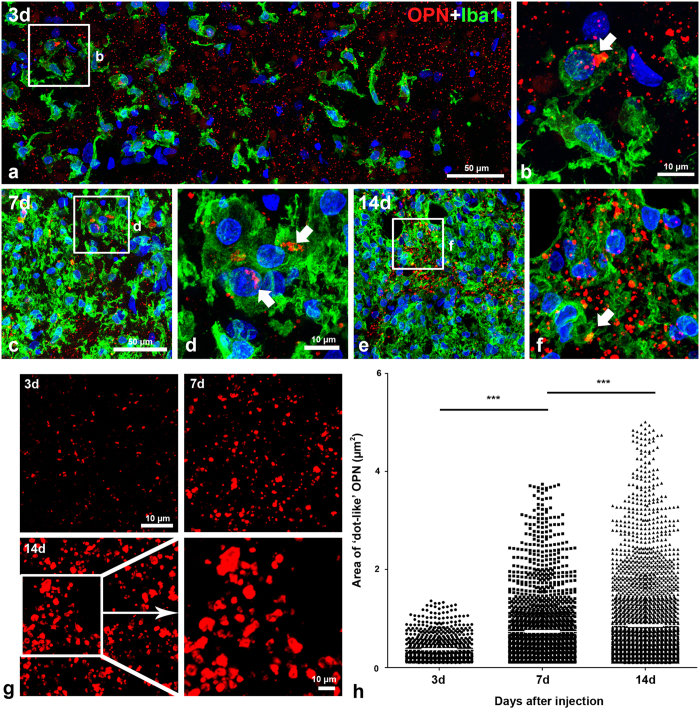
Temporal patterns and quantitative analysis of OPN expression in the 3-NP-injured striatum. (**a**–**f**) Double-labelling with OPN and ionised calcium-binding adaptor molecule 1 (Iba1) 3 (**a,b**), 7 (**c,d**), and 14 days (**e,f**) post-lesion. Note the two different OPN-positive profiles: prominent perinuclear OPN (white arrows in b, d, and f) within brain macrophages, and a punctate pattern of OPN staining in the extracellular matrix. In addition, note that brain macrophages containing perinuclear OPN are rare 3 days post-lesion, but tend to increase by days 7 and 14. (**g**) Temporal morphological features of the dot-like OPN staining after the 3-NP injection. Note that dot-like OPN staining appears to increase in size progressively through day 14, at which time large structures with varying shapes are frequently observed. (**h**) Scatterplot of the cross-sectional areas of the OPN-positive structures, excluding those localised within brain macrophages. Data are presented as means ± standard errors of the mean. Note the progressive increase in the sizes of the OPN-positive structures over the 2-week time period post-lesion. ***P < 0.0001.

We examined the spatiotemporal localisation of OPN-positive staining in the striatal lesions during the experimental period following the 3-NP injection. Double-labelling with OPN and ionised calcium-binding adaptor molecule 1 (Iba1), which labels brain macrophages and microglia, revealed two different patterns of OPN protein expression in terms of localisation and shape. On day 3 post-lesion, OPN-positive staining was visible as small granular dots, most of which appeared to be located in the extracellular matrix. However, some of these dots were localised to the perinuclear region of brain macrophages in a region that may have corresponded to the Golgi complex (Fig. 3a,b). However, this dot-like OPN immunoreactivity was negligible or absent on days at 1 and 2 after the last injection of 3-NP (data not shown). OPN-labelled macrophages were very occasionally visible in the affected striatum on day 3, but were abundant on days 7 (Fig. 3c,d) and 14 (Fig. 3e,f) when the dot-like OPN staining was also diffusely distributed among the brain macrophages.

Careful investigation of the OPN-labelled sections revealed that the dot-like OPN staining appeared to increase in size and number progressively through day 14, at which time large OPN-positive structures with varying shapes were frequently observed. Some of these structures appeared hollow and tube-like (Fig. 3g). This expression pattern was consistent with the results of the quantitative analysis of the time-dependent changes in the sizes of the OPN-positive structures, excluding those localised within Iba1-positive microglia. As shown in Fig. 3h, a progressive increase in the sizes of the OPN-positive structures was observed over the 2-week period following the lesion. The mean cross-sectional area of the OPN staining 14 days after the lesion was 0.86 ± 0.02 μm^2^ (n = 3,114), which was higher than those observed on days 3 (0.37 ± 0.01 μm^2^, n = 834) and 7 (0.74 ± 0.02 μm^2^, n = 1,958) after the lesion. These differences were highly significant (*p* < 0.0001).

### OPN protein localised within mitochondria in degenerating neurites 3 days post-lesion

**Figure 4:**
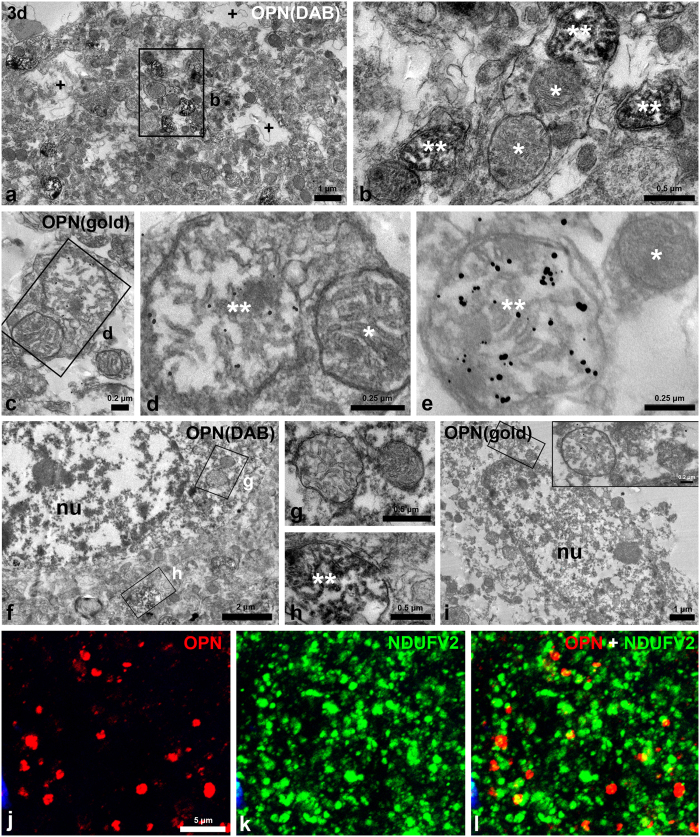
Ultrastructural localisation of OPN within mitochondria of degenerating dendrites 3 days post-lesion. (**a**–**i**) Electron microscopy using immunoperoxidase (**a,b,f–h**) and immunogold/silver methods (**c–e, i**). (**a–e**) OPN protein is specifically localised within swollen mitochondria which have disorganised cristae (** in b, d, and e), but not within normal-appearing mitochondria (* in b, d, and e) of dendrites. Note that vacuoles of different sizes ( + in a), which result from neurite degeneration, are frequently observed. (**f–j**) DAB reaction or silver grains, indicative of OPN, is not specifically associated with any particular organelles including mitochondria (g, inset in i) within the soma of degenerating neurons. Note that prominent OPN is observed within swollen mitochondria (** in h) in the adjacent neurites. nu, nucleus. (b, d, g, h, and inset in i) Higher-magnification views of the boxed areas shown in a, c, f, and i, respectively. (**j**–**l**) Double-labelling for OPN and NADH dehydrogenase flavoprotein 2 (NDUFV2) reveals that OPN-positive dots are co-localised in part with NDUFV2. Cell nuclei appear blue after DAPI staining.

We used combined immunoperoxidase and immunogold/silver staining procedures to further characterise the time-dependent changes in OPN staining in the affected striatum. Three days after the lesion, the lesioned striatum had alterations in the arrangement and morphology of neurites, and displayed many vacuoles of different sizes that were indicative of neurite degeneration (Fig. 4a). At this time point, highly electron-dense diaminobenzidine (DAB) reaction products or silver-enhanced immunogold particles were specifically localised within swollen mitochondria with disorganised cristae in some neurites (Fig. 4b–e). These neurites were determined to be dendrites based on their sizes, shapes, and the presence of mitochondria and microtubules^45^. However, OPN protein was not associated with apparently normal mitochondria (even in the same dendrites), other organelles, or the plasma membranes of dendrites (Fig. 4b–e). In addition, the DAB reaction and immunogold labelling were not specifically associated with mitochondria in the neuronal soma, which showed characteristic features of necrotic cell death, i.e., nuclei with small patches of clumped chromatin and pronounced disintegration of cytoplasmic organelles (Fig. 4f–i). However, OPN protein was prominent within degenerating mitochondria in adjacent neurites (Fig. 4h). In addition, double-labelling for OPN and reduced nicotinamide adenine dinucleotide (NADH) dehydrogenase (ubiquinone) flavoprotein 2 (NDUFV2), which is an enzyme in the inner mitochondrial membrane and is involved in oxidative phosphorylation and proton transport^46^, revealed that OPN-positive dots were indeed partially co-localised with NDUFV2 (Fig. 4j–l).

### OPN protein localised along the membranes of degenerating neurites 14 days post-lesion

**Figure 5:**
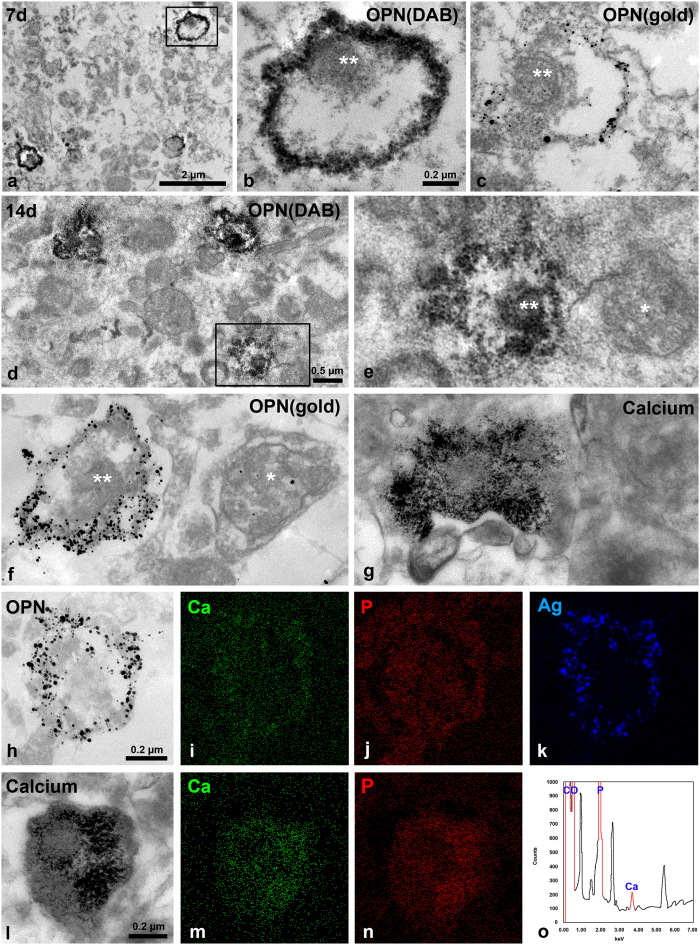
Close spatial correlation between OPN and calcium within degenerating dendrites. (**a**–**c**) OPN immunoreactivity shown by highly electron-dense DAB grains (**a,b**) and silver-enhanced immunogold particles (**c**) is localised along the margins of degenerated neurites 7 days post-lesion. Note that no OPN is detected within the small and highly electron-dense mitochondria (** in b and c) within these neurites. (**d**–**f**) DAB (**d,e**) or silver grains (**f**) for OPNs are accumulated along the surface of degenerating dendrites at 14 days. Note that mitochondria (** in e and f) within dendrites with conspicuous enrichment of OPN are small and electron dense, while adjacent unlabelled neurites contain apparently normal mitochondria (* in e and f). (**g**) Needle-like calcium crystal aggregates (detected using the osmium/potassium dichromate method) are observed within what appears to be degenerating neurites, which are very similar to those of OPN-labelled profiles. (**h–k**) Bright-field transmission electron microscopy (TEM) micrographs showing silver-enhanced immunogold for OPN (**h**) and EDAX elemental mapping of calcium (**i**), phosphorus (**j**) and silver (**k**). Note that the prominent signals for calcium are observed within the OPN-labelled profile, and that silver signals correspond to silver grains indicating OPN. (**l**–**n**) Bright-field TEM micrographs (**l**) and elemental mapping of calcium (**m**) and phosphorous (**n**) using ultrathin section treated with the osmium/potassium dichromate method. Note that calcium-related signals are conspicuous within neurites filled with needle-like crystals. (**o**) X-ray spectrum analysis of i shows the significant peaks for calcium (Ca) and phosphorous (**P**).

Seven days after treatment with 3-NP toxin, DAB grains or silver-enhanced immunogold particles, both of which indicated the presence of OPN protein, were localised along the membranes of degenerated dendrites containing small and highly electron-dense mitochondria. However, no OPN was detected within these mitochondria or the remainder of the dendroplasm (Fig. 5a–c). The ultrastructural features of the OPN-positive staining 14 days post-lesion were similar to those at 7 days, except that the OPN staining at the peripheral outline of dendrites was more intense after 14 days (Fig. 5d–f). An ultrastructural study using the osmium/potassium dichromate method revealed that calcified structures containing rod-like calcium crystals were frequently observed in the affected striatum, and that they were very similar to the OPN-labelled staining patterns in size, shape, and distribution (compare Fig. 5e,f and g). This indicates that these calcified structures may represent OPN-labelled neurites. We further investigated the relationship between OPN and calcium by combining field emission-transmission electron microscopy (FE-TEM) with electron probe microanalysis and immunogold/silver electron microscopy or electron microscopy using the osmium/potassium dichromate method. As shown in Fig. 5h–k, conspicuous calcium-related signals were predominantly localised to the margins of degenerating neurites, which corresponded well with the silver signals indicating the presence of OPN. In addition, the needle-like crystal aggregates in dendritic regions in ultrathin sections treated using the osmium/potassium dichromate method were rich in calcium (Fig. 5l–n). Moreover, calcium and phosphorus peaks were detected in these crystals (Fig. 5o).

### Three-Dimensional analysis of the OPN-positive staining in the lesioned striatum

**Figure 6:**
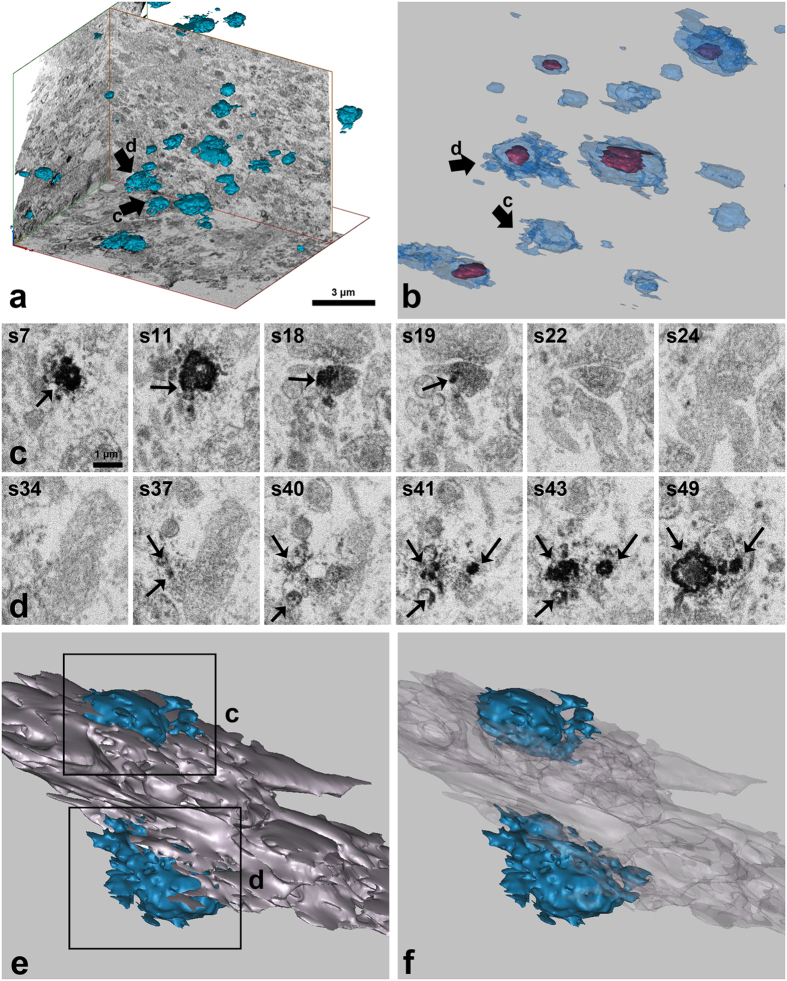
Three-dimensional (3D) reconstructions of the OPN-positive structures. (**a**) 3D distributions of OPN-positive structures (blue) within the affected striatum 14 days post-lesion. The reconstruction is made from a series of 234 consecutive sections using focused ion beam milling (FIB)-scanning electron microscopy (SEM). Note that OPN profiles of variable shapes and sizes are randomly distributed. (**b**) OPN-positive profiles are shown at higher magnification. Note that most OPN profiles (light blue) contain identifiable mitochondria (mahogany). (**c**, **d**) Twelve FIB-SEM images from a series of 60 consecutive sections through two OPN-positive profiles that are marked by arrows in a and b. Note that electron-dense DAB grains for OPN are marked by arrows. (**e**, **f**) 3D reconstruction of two OPN-positive neurites and adjacent structures from 60 consecutive sections containing images shown in c and d. (**f**) Same view as e, but the OPN-negative area is now transparent. Note that one OPN profile (blue, upper part) appears to be directly associated with the major dendrite that is negative of OPN (purple-grey), and the latter is found to be attached to other OPN profile (blue, lower part).

We analysed the shapes and spatial localisations of the OPN-labelled profiles within the affected striatum 14 days post-lesion using FIB-SEM. Morphological tracing and 3D reconstruction were performed in all 234 consecutive sections, which had a total thickness of about 14 microns. We identified 47 OPN-positive structures with a mean volume of 0.52 ± 0.11 μm^3^ in this reconstructed volume. These structures appeared to be randomly distributed within the lesion (Fig. 6a). The shapes and sizes of the OPN-positive structures were variable; some were associated with mitochondria (Fig. 6b). For a detailed analysis of the spatial relationships between the OPN staining pattern and the somatodendritic domains, we traced individual OPN-positive structures and linked them back to the parent dendrites using a series of more than 60 consecutive sections. Three-dimensional reconstruction of some OPN-positive neurites revealed that they appeared to directly extend onto the larger dendrites that were negative for OPN (Fig. 6c–f). These data indicate that OPN-labelled structures are in direct contract with parent dendrites rather than fragmented debris originating from them.

## Discussion

We demonstrated that there is a pronounced induction of OPN expression in the lesioned striatum starting 3 days after the 3-NP injection. This increase in OPN expression was correlated with a pronounced increase in labelling with the cell death marker TUNEL, neuronal degeneration marker FJB, and the loss of DARPP32 immunoreactivity. OPN protein was expressed in a punctate pattern as observed using a light microscope. This staining pattern was scattered throughout the striatal lesion. Our observations are consistent with those made in previous studies of the role of OPN in brain injury, including our own^3^,^23^,^24^,^25^,^47^,^48^,^49^. Immunoblot analysis indicated the presence of OPN protein only in 3-NP-injured striatal tissue. The major OPN band, which corresponds to full-length OPN, was prominent 3–7 days post-lesion, but declined in intensity by day 14. This may be attributable to the creation of a fibrotic scar in the lesion core and the corresponding loss of OPN (Fig. 1a). In addition, smaller molecular-weight OPN proteins (50 kDa or 25 kDa), which have been shown to be cleavage products of matrix metalloproteinase activity^50^,^51^,^52^, were found to be increased in striatal tissue at earlier time points (days 3 and 7). However, the roles and significance of these smaller fragments are unknown. Together these data indicate that distinct patterns of OPN activity are associated with the initiation and progression of 3-NP induced striatal neurotoxicity.

At the subcellular level, OPN exhibited a punctate expression pattern that varied in localisation and shape: larger and more prominent puncta were located around the perinuclear region of brain macrophages, indicating localisation to the Golgi complex^23^. We also observed a scattered pattern of puncta in the extracellular matrix. The latter puncta appeared to increase in number and size over the 2-week post-injury period. Our observations were confirmed using morphometric image analysis. Ultrastructural investigations further indicated that OPN protein had varying spatiotemporal localisation patterns after 3-NP injection. OPN protein accumulates to a great extent along the membranes of degenerating neurites on days 7–14. This is a pattern that was substantially different from the clear intramitochondrial localisation observed in neurons at earlier stages (3 days). Most OPN-labelled structures were recognised as dendrites based on their sizes, shapes, and the presence of mitochondria^45^, although some stained structures were regarded as unidentifiable small neurites.

In addition, the morphological data derived from FIB-SEM and 3D reconstruction revealed that the OPN staining had an appearance reminiscent of fragmented cell debris from degenerating neurites, most of which contained mitochondria. Because the resolution of FIB-SEM images obtained using Epon-embedded samples was not sufficient to clearly distinguish different ultrastructures, we were unable to obtain precise information regarding the detailed spatial relationships between OPN profiles and somatodendritic domains using 3D reconstruction. Nevertheless, our findings provide us with insight into the spatial relationship between OPN-labelled calcified neurites and adjacent neurites. We found that calcified neurites are not merely fragmented dendrites, but are rather in direct contact with OPN-negative, calcification-free larger degenerating neurites. The morphologies of the 3D-reconstructed OPN-positive structures suggest that they might be derived from the dendritic spines of degenerating neurons, although this is less likely because rat neostriatal dendritic spines are smaller in size and do not contain mitochondria or other organelles^53^. Nevertheless, our 3D reconstruction study leaves open the possibility that the OPN-positive structures are either axons or dendritic spines. Thus, more detailed 3D reconstruction analyses at higher resolutions are required to obtain a finely detailed ultrastructural characterisation of the OPN-labelled moieties present during the calcification process.

OPN protein was first detected within swollen and disrupted mitochondria, but was absent from apparently normal mitochondria in degenerating dendrites in the injured striatum. By day 7, however, OPN protein was distinctively expressed along the periphery of degenerating dendrites. This pattern became more evident on day 14. We recently reported that, in a rat model of transient forebrain ischaemia, selective calcium deposits manifest within the mitochondria of degenerating dendrites acutely after reperfusion, although extensive calcification is observed with time^33^. This spatiotemporal progression of calcification after ischaemic insult appears to be very closely comparable to the OPN expression pattern observed in our study. In addition, several lines of inquiry have revealed that intracellular calcification initially occurs in the mitochondria of degenerating neurons and subsequently propagates throughout the whole cell after brain insults^54^,^55^,^56^,^57^. What leads to intramitochondrial OPN expression is not certain, but it can be inferred that OPN is initially localised in degenerating mitochondria where initial intracellular calcification occurs, and then accumulates in the periphery of the degenerating neurites from where calcification subsequently spreads to the entire dendroplasm.

The above scenario is supported by observations made using the osmium/potassium dichromate method, which was used to show that the distribution patterns, shapes, and sizes of the dendritic structures filled with rod-like calcium crystals were all remarkably similar to those of the OPN-labelled structures. Moreover, electron probe microanalysis using calcium-fixated TEM or immune-gold/silver EM was supportive of this hypothesis. However, why OPN was not localised in the remainder of the dendroplasm or even within mitochondria of degenerating dendrites despite being darkened and condensed is unknown and perplexing. One explanation for this unexpected result is that the procedure for immunoelectron microscopy can alter the mineral content and distribution of minerals in the tissue, which may result in insufficient calcium inside dendrites available for binding with OPN. Our observations made using the osmium/potassium dichromate method revealed that the dendrites were filled with calcium crystals, as described above.

Despite several lines of evidence supporting links between intramitochondrial calcification and extracellular calcification progression in response to ischaemic insults 30,31,58,59, scarce information is available regarding the involvement of OPN in the initiation of calcium precipitation, although there is a study of intramitochondrial expression of OPN in a 3-NP model^34^. When systemically administered to rodents, 3-NP selectively damages medium spiny neurons in the striatum via several mechanisms involving excitotoxicity and oxidative stress^60^,^61^,^62^, with all of the mechanisms apparently contributing to the ischaemic insult. Nonetheless, mitochondrial toxicity is the key to 3-NP neurotoxicity, and mitochondrial dysfunction caused by 3-NP may impair calcium ion sequestration and lead to calcium overload^35^,^36^. In addition, 3-NP irreversibly increases intracellular calcium^63^, and injured neurons initially show mitochondrial swelling with disorganised cristae, indicating that early events in 3-NP-induced neurotoxicity involve mitochondrial damage^64^,^65^,^66^,^67^. In this regard, intramitochondrial expression of OPN in the 3-NP model indicates that OPN may act as a potent regulator of mitochondrial calcium homeostasis and/or intracellular calcification process^34^. Alternatively or in addition, OPN may affect mitochondrial morphology and integrity, given a recent study showing that OPN stimulates apoptosis in myocytes via the oxidative stress and mitochondrial death pathway^68^,^69^. Further, study is needed for better and more detailed knowledge the intramitochondrial functions of OPN.

We observed the rare expression of OPN in the perikarya and proximal dendrites of dying or dead cells, and even within their disrupted mitochondria, even though these structures exhibit the classical features of distinct cell death, i.e., the disintegration of membranes and cytoplasmic organelles. This might be explained in part by the low calcium levels in these areas, as suggested previously^24^,^25^. It has also been widely accepted that dendritic and axonal morphological changes, including calcium deposition, occur in advance of neuronal somatic changes in various neurodegenerative diseases^56^,^70^,^71^. In fact, a recent study has shown that dendrites of cortical neurons, but not their somatic regions, are more susceptible to oxidative stress and excitotoxic injury^72^. Consistent with the above findings, our recent data revealed that the dendrites, rather than the somata, of degenerating neurons are prone to calcification after ischaemic insults, suggesting that dendritic mitochondria may be the first to respond to the calcium overload elicited by ischaemic insults^33^. Moreover, our 3D reconstruction analysis revealed a clear preference for OPN accumulation in distal neuritis vs. proximal large-calibre dendrites or neuronal cell bodies. Considering these findings together, we speculate that calcium precipitation within distal dendrites provides a matrix for the binding of OPN. However, we cannot rule out that limitations in the immunohistochemical detection of OPN protein might have precluded us from recognizing very early cellular events, or that detectability might even have been masked by the pronounced disintegration of cellular organelles. Whatever the mechanisms involved, our data suggest that intramitochondrial OPN is, at least partly, attributable to initial calcium precipitation within degenerating dendrites that are prone to calcification.

Another interesting aspect of our data was the possibility that the source of OPN protein might have been multicellular in the affected striatum. At 3 days post-lesion, only a few brain macrophages containing OPN in their Golgi region were visible in the striatal lesion, with massive infiltration into the lesion being clearly observed by 7 days, which parallels the time course of increased OPN expression. These results indicate that brain macrophages synthesize and secrete OPN protein, which accumulates on the surface of the degenerated dendrites, as suggested previously^23^,^25^. However, the tiny granular OPN protein, localised in dendritic mitochondria by EM, appeared profusely at 3 days post-lesion, even prior to brain macrophages infiltrating into the lesion. These findings suggest that brain macrophages are not the only cellular source of OPN in striatal lesions. OPN has been reported to be produced by degenerating neurons in several neuropathological disorders^47^,^73^,^74^,^75^,^76^, and by cortical neurons in HIV-associated associated neurocognitive disorders^77^. The presence of intracellular OPN, which lacks the vesicle-targeting signal sequence, has been reported in various types of cells including activated macrophages, osteoclasts and fibroblasts, and it has been implicated in a number of cellular processes, including migration, fusion, and motility^78^,^79^,^80^,^81^,^82^. Notably, Baliga *et al*. proposed that the intracellular form of OPN is increased in the cytoplasm of the cortex during early cerebral ischaemia-reperfusion, suggesting a role of OPN as a responder to stroke-induced cell damage^83^. Thus, we speculate that degenerating neurons could be the initial source of the OPN protein localised within dendritic mitochondria, but it remains to be established whether this indeed reflects the presence of intracellular OPN.

In conclusion, our study describes a sequential induction pattern of OPN expression in the rat striatum during 14 days after 3-NP injection. The early induction of OPN protein specifically within dendritic mitochondria was followed by the profuse accumulation on the surface of degenerating dendrites on days 7–14 post-injection. The time-dependent localisation of OPN protein is likely to correlate with the spatial profile of intracellular and extracellular calcification progression after brain insults. Our data suggest that OPN may play an important role in the initiation and progression of the microcalcification that occurs in the striatum in response to brain insults.

## Methods

### Animal Preparation

All experimental procedures were conducted in accordance with the Laboratory Animal Welfare Act, the Guide for the Care and Use of Laboratory Animals, and Guidelines and Policies for Rodent Survival Surgery, and were approved by the Institutional Animal Care and Use Committee at the College of Medicine, The Catholic University of Korea (Approval Number: CUMC-2014-0006-01). All efforts were made to minimise animal suffering and to reduce the number of animals used.

Fifty-four adult male Sprague-Dawley rats (250–300 g) were used in this study. Animals were housed in groups of three per cage in a controlled environment at a constant temperature (22 ± 5 °C) and humidity (50 ± 10%) with food (gamma ray-sterilised diet) and water (autoclaved tap water) available *ad libitum*. They were maintained on a 12-hour light/dark cycle. 3-NP (Sigma-Aldrich, St. Louis, MO, USA) was dissolved in buffered saline (pH = 7.0) and administered intraperitoneally (i.p.) at a dose of 15 mg/kg once daily for 3 days. All 3-NP-injected rats were evaluated daily for the presence of behavioural deficits, and only rats exhibiting neurological deficits, such as hind limb impairment or kyphotic posture, recumbency, and impaired postural adjustments were included in the experimental group^84^.

Animals were sacrificed 1, 2, 3, 7, and 14 days after the final injection of 3-NP (n = 6 rats for each time point). The control group (n = 3) received intraperitoneal injections of the same volume of normal saline for 3 consecutive days. The rats in this group were sacrificed 3 days after the final injection. We used experimental rats 3, 7, or 14 days after the last injection of 3-NP (*n* = 3 per group) for immunoelectron microscopic analyses. The animals were perfused transcardially with 4% paraformaldehyde in 0.1 M phosphate buffer (PB, pH 7.4) after anaesthesia using 10% chloral hydrate (4 mL/kg i.p.). The brain tissues were equilibrated with 30% sucrose in 0.1 M PB and frozen fully for light microscopic study.

For the immunoblot analyses, rats from 4 groups (controls, and experimental rats sacrificed 3, 7, or 14 days after the last injection of 3-NP) (*n* = 3 per group) were killed by decapitation under anaesthesia (10% chloral hydrate; 4 mL/kg i.p). The cortical and striatal tissues were carefully dissected under a stereoscopic microscope and immediately frozen in liquid nitrogen. Brain samples were stored at −70 °C until further processing.

### Immunoblot analysis

For the immunoblot analysis, tissues were homogenised and protein was isolated from the cortex or striatum using boiling lysis buffer (1% sodium dodecyl sulphate [SDS], protease inhibitor cocktail [Roche, Mannheim, Germany], phosphatase inhibitor cocktail [Roche], and 10 mM Tris; pH 7.4). Equal amounts (15 μg) of total protein was separated by SDS polyacrylamide gel electrophoresis (10%) and transferred to polyvinylidene difluoride membranes. The membranes were blocked in 5% bovine serum albumin (Sigma-Aldrich) in Tris-buffered saline for OPN and in 0.1% Tween-20 and 5% skim milk for β-actin. We used a mouse monoclonal anti-rat OPN antibody (American Research Products, Belmont, MA, USA; 1:2,500) and anti-β-actin (Sigma-Aldrich; 1:20,000). The membranes were then incubated with peroxidase-coupled secondary antibodies (Millipore, Temecula, CA, USA; 1:1,000) for 1 hour at room temperature. The blots were visualised using electrochemiluminecent western blotting substrate (Promega, Madison, WI, USA).

### Tissue preparation and immunohistochemistry

For OPN immunohistochemistry, coronal cryostat sections (25-μm thick) were incubated overnight at 4 °C with a mouse monoclonal anti-rat OPN antibody (American Research Products, 1:300). Primary antibody binding was visualised using peroxidase-labelled goat anti-mouse antibody (Millipore; 1:100) and 0.05% 3,3′-diaminobenzidine tetrahydrochloride with 0.01% H_2_O_2_ as a substrate. The specificity of OPN immunoreactivity was confirmed by the absence of immunohistochemical staining in sections from which the primary or secondary antibody had been omitted. Tissue sections were scanned and photographed using a slide scanner (SCN400, Leica Microsystems Ltd., Mannheim, Germany). Images were converted to TIFF format, and contrast levels adjusted using Adobe Photoshop v. 10.0 (Adobe Systems, San Jose, CA, USA).

For double-immunofluorescence histochemistry, the sections were incubated at 4 °C overnight with a mix of a mouse monoclonal anti-rat OPN antibody (1:300) and a primary rabbit polyclonal antibody to Iba1 (Wako Pure Chemical Industries, Ltd., Japan; 1:500) or DARPP-32 (Cell Signalling Technology, Danvers, MA, USA; 1:200) or NDUFV2 (Proteintech, Rosemont, IL, USA; 1:100). Antibody staining was visualised using Cy3-conjugated goat anti-mouse antibody (Jackson ImmunoResearch, West Grove, PA, USA; 1:2,000) and Alexa Fluor 488 goat anti-rabbit antibody (Thermo Fisher, Waltham, MA, USA; 1:300). Control sections were prepared as described above. Counterstaining of cell nuclei was carried out using DAPI (4′,6-diamidino-2′-phenyindole, Roche; 1:2,000) for 10 minutes. Slides were viewed with a confocal microscope (LSM 700; Carl Zeiss Co. Ltd., Oberkochen, Germany) equipped with four lasers (Diode 405, Argon 488, HeNe 543, HeNe 633). Images were converted to the TIFF format, and contrast levels were adjusted using Adobe Photoshop v.10.0.

To simultaneously detect apoptotic cells and OPN and DARPP-32, we performed triple-labelling using TUNEL staining with double-immunofluorescence for OPN and DARPP-32. Free-floating sections (25-μm thick) were stained using the TUNEL method according to the manufacturer’s protocol (Roche Diagnostics Corporation, Indianapolis, IN, USA). This was followed by a 1-hour incubation with Alexa Fluor 488-conjugated goat anti-mouse antibody (Thermo Fisher; 1:300), Alexa Fluor 647 goat anti-rabbit antibody (Thermo Fisher; 1: 300), and Cy3-conjugated streptavidin (Jackson ImmunoResearch; 1:2,000) for TUNEL staining. Counterstaining of cell nuclei was carried out for 10 minutes using DAPI.

For histologic evaluation of degenerating neurons and their relationships with OPN expression, we used FJB histochemistry and immunohistochemistry for DARPP-32 or osteopontin on sequential cryostat sections. For FJB staining, sections were stained with 0.0004% FJB (Millipore) in distilled water containing 0.01% acetic acid for 30 minutes according to the manufacturer’s protocol. After rinsing in distilled water, the sections were immersed in xylene and cover-slipped with the DPX mounting medium (Sigma-Aldrich). For immunohistochemistry, serial sections were incubated at 4 °C overnight with a mouse monoclonal anti-rat OPN or a rabbit polyclonal anti-DARPP-32.

### Immunoelectron microscopy, osmium/potassium dichromate staining, and electron probe microanalysis

We used pre-embedding for immunoperoxidase and immunogold/silver staining experiments. For immunoperoxidase staining, floating vbratome sections (50-μm thick) were immunostained with a mouse monoclonal anti-rat OPN antibody. After post-fixation, dehydration, and embedding in Epon 812, areas of interest were excised and glued onto resin blocks. Ultrathin sections (70–90-nm thick) were cut and observed under an electron microscope (JEM 1010, JEOL, Tokyo, Japan) with slight uranyl acetate staining. When using the pre-embedding immunogold/silver technique, vibratome sections were incubated with the primary antibody as above. The sections were then incubated with an anti-mouse secondary antibody conjugated to 1-nm gold particle-tagged nanogold (Nanoprobes, Stony Brook, NY, USA; 1:100) for 2 hours. Silver enhancement was performed using the HQ silver enhancement kit (Nanoprobes) for 3 minutes. Ultrathin sections were prepared as described above and observed using an electron microscope.

We used the osmium/potassium dichromate method of Probst^38^ to detect calcium precipitates at the ultrastructural level. Vibratome sections (50-μm thick) were post-fixed in 1% osmium tetroxide (OsO_4_) and 2.5% potassium-dichromate (K_2_Cr_2_O_7_; Hayashi Pure Chemical Industries Ltd., Osaka, Japan) for 1 hour at 4 °C. After dehydration and embedding in Epon 812, areas of interest were excised and glued onto resin blocks as described above. Ultrathin sections were lightly stained with uranyl acetate and observed with an electron microscope (JEM-1010).

For electron probe microanalysis, thin sections (80–100 nm) of selected regions were placed on copper grids and carbon-coated (approximately 20 nm in thickness). The chemical compositions of the silver-enhanced immunogold-labelled structures were analysed using an FE-TEM (JEM-2100F, JEOL) equipped with EDAX (for elemental detection by X-ray analysis) installed at the Korea Basic Science Institute. The operative voltage was 200 kV.

### Quantitative analysis

To quantify the time-dependent changes in the dot-like OPN-positive structures in the striatum in rats subjected to 3-NP treatment, the cross-sectional areas of these profiles obtained using confocal microscopy were analysed using TOMORO ScopeEye 3.6 software (JNOPTIC Co., Seoul, Korea). Sections double-labelled for OPN and Iba1 in experimental rats 3, 7, and 14 days after 3-NP injection (*n* = 3 per time point) were obtained from the invariable region between bregma levels 0.24 mm and 1.56 mm dorsal^85^. Three areas (50 × 50 μm per field) in the lesion core of each section were chosen, and captured at 1,260x magnification under constant viewing conditions. Images were acquired at a resolution of 512 × 512 pixels, and those signals larger than 5 pixels were considered to be positive. All OPN-positive staining, excluding staining localised within Iba1-positive microglia, were counted, and their cross-sectional areas were measured in square micrometres. The total area quantified was kept constant for each section analysed. The differences in size between groups were assessed with two-tailed unpaired Student’s t-test, using Excel 2013 (Microsoft, Bellevue, WA, USA) and GraphPad Prism, version 5 (GraphPad Software Inc., San Diego CA). Differences with *P* values of less than 0.05 were considered statistically significant.

### Serial section-based 3D reconstruction

For 3D reconstruction, brain tissue samples were obtained from Epon-embedded DAB-labelled slices from rats subjected to 3-NP toxin 14 days after injury, and prepared as described above. The area of interest (19.69 μm x 18.27 μm) was selected and 234 consecutive serial sections (60-nm thickness) were imaged using FIB-SEM (1540XB, AURIGA, Carl Zeiss, Germany) incorporating a gallium liquid metal ion source and *in-situ* SEM with a thermal field emission source. Individual OPN-positive structures, mitochondria, and adjacent structures were manually traced with different colours through the stacks of images using Adobe Photoshop. We then aligned each section image. The marked structures were masked and exported to the 3D modelling program Mimics 19.0 (Materialise, Leuven, Belgium), along with information regarding slice thickness, actual pixel size, and orientation of the image. Volumes of reconstructed OPN-positive structures were also calculated using Mimics 19.0. To visualise the OPN-positive structures within the original EM images, the 3D-reconstructed profiles were stacked onto the reconstructed EM images. In addition, the OPN-positive structures or mitochondria, and their spatial relationships to adjacent neurites, were visualized in 3D reconstructions by making adjacent neurites or OPN-positive structures transparent.

## Additional Information

**How to cite this article:** Riew, T.-R. *et al*. Spatiotemporal expression of osteopontin in the striatum of rats subjected to the mitochondrial toxin 3-nitropropionic acid correlates with microcalcification. *Sci. Rep.* **7**, 45173; doi: 10.1038/srep45173 (2017).

**Publisher's note:** Springer Nature remains neutral with regard to jurisdictional claims in published maps and institutional affiliations.
